# Burden and Impact of Drug Shortages in a Saudi Tertiary Hospital: A Single-Center Cross-Sectional Survey

**DOI:** 10.3390/healthcare14101359

**Published:** 2026-05-15

**Authors:** Njoud Altuwaijri, Fai Alkathiri, Rihaf Alfaraj, Mohammed A. Aljallal, Abrar S. Abduljawad, Asmaa K. Alzhrani, Najd B. Alnassar, Amenah Alkaf, Sarah O. Abaalola, Omamah Eid, Fahad I. Al-Jenoobi

**Affiliations:** 1 Department of Pharmaceutics, College of Pharmacy, King Saud University, Riyadh 11451, Saudi Arabia; naltuwaijri@ksu.edu.sa (N.A.); ralfaraj@ksu.edu.sa (R.A.); 445920422@student.ksu.edu.sa (A.S.A.); 445920426@student.ksu.edu.sa (A.K.A.); alnassarnajd@gmail.com (N.B.A.); alkafamenah@gmail.com (A.A.); saraomar.ab@hotmail.com (S.O.A.); 445205574@student.ksu.edu.sa (O.E.); aljenobi@ksu.edu.sa (F.I.A.-J.); 2 Saudi Food and Drug Authority, Riyadh 13513, Saudi Arabia; majallal@sfda.gov.sa

**Keywords:** essential medicines, drug shortages, Saudi Arabia, Saudi Food and Drug Authority, healthcare system, patient outcomes, supply chain management

## Abstract

**Background:** Drug shortages represent a growing challenge to healthcare systems worldwide, affecting treatment continuity and patient outcomes. This study assessed the burden and perceived impact of drug shortages from both healthcare professionals’ and patients’ perspectives in a Saudi tertiary hospital. **Methods:** A cross-sectional survey was conducted in April 2025 at King Abdulaziz Medical City, Riyadh, Saudi Arabia. Convenience sampling was used to recruit healthcare professionals with at least two years of experience and adult outpatients. Structured questionnaires assessed shortage frequency, affected drug classes, perceived impacts, and management practices. The findings were descriptively analyzed and compared with the Saudi Food and Drug Authority (SFDA) national shortage data for the corresponding 12-month period. **Results:** A total of 230 healthcare professionals and 243 patients participated. Among healthcare professionals, 89.1% reported experiencing at least one drug shortage, with 38.3% encountering shortages more than ten times annually. Anti-infectives (36.5%) and analgesics (35.7%) were the most frequently reported classes. The most common response was prescribing alternative medications (77.4%), with 55.3% perceived as adequately effective and 30.8% as less effective. Delayed care was the most frequently reported consequence (44.0%). Among patients, 30.9% reported experiencing shortages, 46.7% reported some degree of health impact, and 28.1% incurred additional costs. Awareness and utilization of the SFDA reporting system were low in both groups. Comparison with SFDA data revealed discrepancies between hospital-reported and nationally reported shortages. **Conclusions:** Drug shortages were frequently reported and associated with perceived clinical and economic consequences. Gaps between hospital experiences and national reporting highlight limitations in current surveillance systems. Strengthening reporting mechanisms, communication, and supply chain coordination may improve the management of drug shortages.

## 1. Introduction

The availability of essential medicines is a critical determinant of public health outcomes, particularly in regions with diverse socioeconomic landscapes. Saudi Arabia has made significant strides in its healthcare infrastructure since the foundation of the Kingdom in 1932; the government launched numerous initiatives to improve healthcare access and quality, which led to the establishment of the Ministry of Health in 1950. By the 1970s and 1980s, Saudi Arabia had developed a robust healthcare system characterized by a network of hospitals and clinics, alongside the establishment of national pharmaceutical manufacturing capabilities [1].

Drug shortages have been widely reported across healthcare systems worldwide, posing significant challenges to patient care and healthcare delivery. In Saudi Arabia, the prevalence of drug shortages has increased substantially; between 2017 and 2020, 1082 drugs were reported to the SFDA as experiencing shortages, averaging 271 shortage incidents per year and peaking at 544 incidents in 2019 alone, with the COVID-19 pandemic further exacerbating the situation [2]. These shortages have affected several critical therapeutic classes, including antineoplastic, antibiotic, immunosuppressant, gastrointestinal, emergency, respiratory, anesthetic, ophthalmic, psychotropic, and cardiovascular agents. The types of medications in shortage often include different dosage forms of both generic and branded drugs [3].

The sources of these shortages are multifaceted. Supply chain disruptions, exacerbated by global events such as the COVID-19 pandemic, have significantly impacted the availability of medications [1]. Manufacturing problems such as quality control, production, and regulatory affairs issues have also been cited as common causes of drug shortages [1,4]. Regulatory challenges, including stringent and long approval processes, further complicate the situation [4].

The impact of drug shortages on patient care is profound and multidimensional. Delayed treatments due to the unavailability of essential medications can lead to worsening health outcomes. For instance, patients with chronic diseases may experience exacerbations of their conditions, leading to increased morbidity and mortality [5]. A study by Almutairi et al. found that 62% of healthcare providers’ reported drug shortages resulted in delayed patient care, with 45% noting that patients’ health deteriorated as a result [4].

Moreover, certain populations are particularly impacted by the consequences of drug shortages. Elderly patients, who often require multiple medications for chronic conditions, are at increased risk of adverse health outcomes when essential drugs are unavailable [6]. Low-income communities with limited access to alternative treatments also face significant barriers to care [7].

In addition to adverse health outcomes, the economic implications of drug shortages are sometimes substantial. Increased healthcare costs arise from the necessity of using alternative treatments, which may not be as effective or may require additional interventions [7]. A report by the World Health Organization (WHO) indicates that drug shortages can lead to an estimated increase of 20% in healthcare expenditure, primarily due to the use of substitute therapies and emergency care [8].

To improve the stability of drug supply and reduce drug shortages, the Saudi Food and Drug Authority (SFDA) has developed a strong reporting and monitoring system. Companies must report stock levels and expected shortages through the Saudi Drug Information System (SDI). The SFDA also publishes an online drug-shortage list, which is updated regularly and helps healthcare providers to stay informed [9]. In October 2025, the SFDA launched an AI-powered model to predict drug shortages using historical data [10]. This smart system was introduced during the Global Health Exhibition and supports faster responses and better planning, aligning with Saudi Arabia’s Vision 2030 to advance digital health transformation and enhance the efficiency of the national health system [11].

The burden of drug shortages on healthcare providers and systems is also considerable. Healthcare systems must assign additional resources to manage shortages, including time spent on sourcing alternative medications and coordinating [12]. This can lead to increased workloads, stress, and burnout among healthcare professionals, further exacerbating the challenges faced by the healthcare system [5].

Drug shortages also have significant psychosocial effects on patients. The anxiety and stress associated with the uncertainty of obtaining necessary medications can lead to decreased quality of life [13]. Patients may experience feelings of helplessness and frustration, particularly when faced with chronic illnesses that require consistent medication management [5].

Moreover, recurring drug shortages can weaken trust in the healthcare system. Patients may become disappointed with healthcare providers if their needs are not met [14]. This loss of trust can have long-lasting effects on patient–provider relationships and overall health outcomes.

Awareness of the challenges associated with drug shortages is crucial for healthcare professionals, policymakers, and regulatory bodies. However, research indicates that many healthcare providers are often unaware of the extent of drug shortages and their potential impact on patient care [15]. This lack of awareness can lead to inadequate preparation and response strategies, ultimately affecting patient treatment outcomes.

Furthermore, studies have shown that communication between manufacturers, healthcare providers, and regulatory agencies is often insufficient. A survey conducted by the American Society of Health-System Pharmacists (ASHP) found that healthcare providers frequently reported being unaware of impending shortages until they occurred, highlighting the need for improved communication channels [16].

The role of education and training in enhancing awareness of drug shortages is also emphasized in the literature. Educational interventions targeting healthcare providers can increase their understanding of the causes and consequences of drug shortages, as well as the importance of timely reporting to regulatory bodies such as the SFDA [7].

Despite the growing body of literature documenting the prevalence and causes of drug shortages in Saudi Arabia, important gaps remain. Most existing studies have focused on either healthcare professionals or patients in isolation, limiting a comprehensive, system-level understanding of the impact of shortages. There is a lack of research that simultaneously integrates both provider and patient perspectives within a single institutional setting. In addition, few studies have examined the alignment between locally perceived shortages and nationally reported data. Specifically, benchmarking of hospital-based, self-reported shortages against the Saudi Food and Drug Authority (SFDA) national shortage list remains underexplored. Addressing these gaps is essential to better understand discrepancies between frontline experiences and official reporting systems and to inform more effective mitigation strategies.

The primary objective of this study is to assess the perceived burden of drug shortages at a tertiary hospital in Saudi Arabia, as reported by healthcare professionals and patients, with a focus on shortage frequency, affected drug types, and clinical and economic consequences. Both groups were included to provide complementary perspectives: healthcare professionals offer insight into clinical consequences, management challenges, and system-level gaps, while patients provide a direct account of how shortages affect medication access, treatment continuity, and overall well-being. The secondary objectives are to explore patients’ experiences with medication access and treatment continuity during shortages; evaluate healthcare professionals’ awareness of and preparedness for drug shortages; identify communication gaps between healthcare providers, institutions, and regulatory bodies; and propose practical mitigation strategies and policy interventions to address future shortages.

## 2. Methods

### 2.1. Study Design and Settings

This study employs a cross-sectional survey design aimed at assessing the perceived characteristics, experiences, and awareness of healthcare professionals and patients regarding drug shortages within one hospital in Saudi Arabia. The survey was conducted in a major city, Riyadh, specifically targeting healthcare professionals and patients at Riyadh King Abdulaziz Medical City. The hospital is a large, government-funded healthcare system with a total bed capacity of approximately 5421 beds distributed across multiple medical campuses. It operates as a tertiary care institution, providing a comprehensive range of specialized and sub-specialized services, including advanced clinical care, academic programs, and research activities. The hospital also serves as a major referral center, delivering integrated healthcare that spans from primary care to highly specialized tertiary services for National Guard personnel, their dependents, and other eligible patients across various regions in Saudi Arabia. The data collection period was conducted over a four-week period in April 2025, representing a defined data collection period aligned with the study schedule.

### 2.2. Sampling Procedure

The study sample was obtained using a convenience sampling technique. A questionnaire was distributed to patients and all healthcare professionals at the target hospital. Participants were recruited in person. Healthcare professionals were approached within their clinical settings, while patients were recruited during hospital visits and asked to complete the questionnaire on-site. The questionnaire was developed by researchers to evaluate the characteristics of drug shortages within their hospital and to explore healthcare professionals’ experiences and awareness regarding these shortages. Due to convenience sampling and in-person recruitment, the number of individuals approached and those who declined to participate were not systematically recorded; therefore, a formal response rate could not be calculated. Healthcare professionals eligible for inclusion were those currently practicing at King Abdulaziz Medical City in Riyadh, Saudi Arabia, involved in patient care, and with at least two years of professional experience. This criterion was established to ensure that respondents had sufficient clinical exposure to drug-shortage events and familiarity with institutional substitution and procurement protocols. It also ensured their ability to make informed assessments of shortage frequency and patient impact, competencies that are unlikely to be adequately developed within the first two years of practice.

For patients, inclusion was limited to adult outpatients aged 18 years and older who were able to read and understand Arabic or English and complete the questionnaire independently without assistance, to avoid interviewer bias. Patients with cognitive impairments or communication difficulties were excluded to ensure response reliability. Outpatients were specifically selected as they represent individuals actively accessing healthcare services and obtaining medications and, therefore, are more likely to have direct and recent experience with medication availability and shortages in routine care settings. An informed consent statement was presented on the first page of the questionnaire, allowing participants to either proceed with the survey or withdraw at that moment. The study objectives were clearly stated to ensure participants understood the significance of their involvement, with completion of the survey being considered as consent to participate.

### 2.3. Questionnaire Tool

The questionnaire on the impact of drug shortages aims to understand the effects of essential medicine shortages on both patients and healthcare professionals. Participation is voluntary, and the responses remain confidential, contributing to improvements in medication availability. The questionnaire was developed based on a comprehensive literature review investigating various aspects of drug shortages, including their frequency, impact on patient care, and potential solutions [5,17]. It includes an introduction section that gathers demographic information such as age, gender, chronic health conditions, and current prescription medications for patients, while healthcare professionals provide details like professional titles and years of experience. The questionnaire was developed in both English and Arabic and was presented to participants in both languages. To minimize potential sources of bias, the questionnaire was designed with clear and structured items and underwent pilot testing to ensure clarity and consistency. Participation was voluntary and anonymous to reduce response bias, and questions were framed to capture recent and relevant experiences to help limit recall bias.

For patients, the first section assesses experiences with drug shortages over the past 24 months, including the frequency of shortages and their impact on health and well-being. The second section focuses on access to alternative medications, asking whether participants were prescribed alternatives, the time taken to find them, and their perceived quality and effectiveness. The third section assessed the self-reported impact of drug shortages on patients’ overall health, using an ordinal scale, while the fourth section addresses any financial implications, including additional costs incurred. Finally, the questionnaire assesses patients’ awareness of the reasons behind drug shortages and the importance of communication from healthcare providers.

For healthcare professionals, the questionnaire addresses the frequencies and types of shortages encountered, potential adverse events, increased hospitalization rates, and management procedures in place at their facilities. It also seeks insights into expectations for advance notice of shortages and the average duration for resolving these issues, while inviting participants to identify perceived causes and propose strategies to mitigate these challenges. This structured approach aims to gather comprehensive insights into the challenges faced by both patients and healthcare providers due to drug shortages. The questionnaire includes multiple-choice questions to provide a comprehensive understanding of the challenges related to drug shortages.

Finally, drug shortages are monitored nationally through the Saudi Food and Drug Authority (SFDA), which publishes shortage notifications/lists. SFDA entries were mapped to the therapeutic classes used in this study using product name and/or active ingredient information. SFDA-published drug-shortage entries were extracted from the SFDA shortage/anticipated shortage list for the same 12-month recall period as the survey (1 April 2024 to March 2025). Entries were included if the shortage start date fell within the defined window. These data were used for benchmarking purposes to compare hospital-reported shortages with nationally reported shortages and to identify potential discrepancies between local experiences and official surveillance data. SFDA data were mapped to the same therapeutic classes used in the survey and descriptively compared with healthcare professional-reported shortages, without statistical merging, to identify patterns and discrepancies between local and national data.

#### 2.3.1. Validity of Questionnaire Tool

##### Pilot Study

The questionnaire tool was reviewed and validated by two pharmacists. They were asked about the clarity and comprehensibility of the questions, as well as their face validity and whether any of the questions were difficult to understand. Their feedback indicated that the questionnaire was simple to comprehend and complete. Furthermore, before adopting the questionnaire on a broader scale, a pilot study was conducted with a small number of participants (n = 20) to assess its comprehension. Participants confirmed that the questionnaire was clear and straightforward.

##### Content and Clarity Validity Assessment

A panel of five content experts were recruited through purposive sampling. Experts were faculty members and educational researchers with experience in biotechnology, pharmacy practice, and pharmaceutics. Each expert was provided with a questionnaire, a structured content and clarity rating form, and instructions to rate each item independently. Content and clarity validity were calculated using the Item-Level Content Validity Index (I-CVI) and Scale-Level Content Validity Index (S-CVI). The overall scale is S-CVI > 0.80, which is considered to have good content and clarity validity.

### 2.4. Sample Size Calculation and Statistical Analysis

The study setting included 19,935 healthcare professionals during the study period, serving an estimated annual patient population of approximately 2,045,518, with a total bed capacity of about 5428 beds. The minimum required sample size was determined to be 377 healthcare professionals and 384 patients, using a 95% confidence interval, a standard deviation (SD) of 0.5, and a margin of error of 5%. Although the calculated minimum sample size was not fully achieved in both cohorts, the achieved samples (230 healthcare professionals and 243 patients) remain adequate for the descriptive objectives of this study. However, this may affect the precision of estimates and limit broader generalizability. The sample size was calculated using the standard formula:

$$n=\;\frac{z^{2}\;\times p\;(1-p)}{e^{2}}$$

where *Z* = 1.96 for a 95% confidence level, *p* = 0.5, and *e* = 0.05.

Data were analyzed using SPSS Statistics (version 29; IBM Corp., Armonk, NY, USA). Descriptive statistics were used to summarize participant characteristics, with frequencies and percentages reported for all categorical variables. For ordinal variables measured using a 4-point scale (no impact, minor, moderate, or significant) and Likert-type items on healthcare professionals’ perceptions, including advance notice of shortages, resolution time, and effectiveness of implemented strategies, frequency distributions and median values were reported using the original response categories without grouping. Chi-square goodness-of-fit tests were applied to assess whether the observed distributions of categorical variables including age, gender, professional title, years of experience, and shortage frequency, differed significantly from the expected distributions. Multiple-response questions were analyzed using multiple-response frequency analysis, with percentages calculated based on the total number of respondents. A *p*-value of <0.05 was considered statistically significant. Given the descriptive nature of this study, no multivariable modeling or adjustment for confounding variables was performed. Subgroup analyses were not conducted beyond descriptive stratification. Missing or incomplete responses were handled using pairwise exclusion, with analyses performed based on the available data for each variable.

### 2.5. Ethical Approval and Consent to Participate

The study protocol was reviewed, and ethical approval was granted by King Abdullah’s International Medical Research Center—Ministry of National Guard Health Affairs (MNGHA), Saudi Arabia (Reference No: NRR25/085/1). All participants gave their consent for participation. This study was conducted in accordance with the principles of the Declaration of Helsinki. All data was collected anonymously, and no identifiable personal information was obtained. Responses were stored securely, accessed only by the research team to ensure confidentiality and data protection, and retained solely for the purposes of this study in compliance with applicable data protection regulations.

### 2.6. Questionnaires/Data Sheets

The appendices include supporting study documents. Appendix A includes the healthcare professionals’ questionnaire, and Appendix B includes the healthcare users’ questionnaire.

## 3. Results

### 3.1. Healthcare Professionals

#### 3.1.1. Demographic Characteristics

A total of 230 participants were enrolled in this study (Table 1). The majority were female (69.6%), a proportion significantly higher than for males (30.4%), with a gender distribution significantly different from the expected equal distribution (*p* < 0.001).

Nearly half of the respondents were 30–39 years old (48.7%), followed by those younger than 30 years (28.7%) and 40–49 years (16.1%), with only a small fraction aged 50 years or older (6.5%). The distribution of age categories was highly skewed and significantly different from uniform expectations (*p* < 0.001).

In terms of professional experience, participants were relatively evenly distributed between those with 2–5 years (37.4%) and more than 10 years (37.8%) of experience, while 24.8% reported 6–10 years. A significant difference from the equal distribution was observed (*p* = 0.022). Regarding profession, nurses constituted the largest group (40.9%), followed by pharmacists (20.9%), physicians (11.3%), and other healthcare professionals (26.9%). This distribution differed markedly from an equal distribution among professions (*p* < 0.001).

#### 3.1.2. Frequency and Types of Shortages

**Figure 1:**
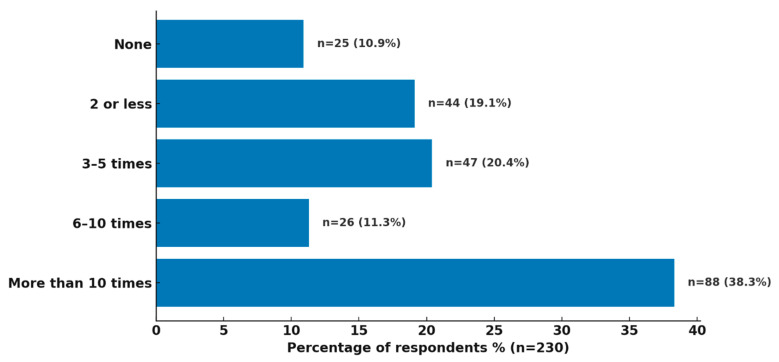
Frequency of drug shortages reported by healthcare professionals (2023–2024).

A total of 230 healthcare professionals responded to the question regarding the frequency of drug shortages experienced over the past year (Figure 1). A large number of respondents (n = 88, 38.3%) reported encountering drug shortages more than 10 times. Meanwhile, 26 respondents (11.3%) experienced 6–10 instances of shortages, followed by 47 respondents (20.4%) who experienced shortages 3–5 times and 44 (19.1%) who experienced 2 or fewer. Notably, only 25 respondents (10.9%) reported no drug shortages during the period, indicating the widespread prevalence of this issue.

Respondents reported a wide range of durations for essential drug shortages (Table 2). A substantial proportion indicated that shortages typically lasted more than a week but less than a month (n = 76, 33.0%). This was closely followed by shortages lasting one week or less (n = 74, 32.2%) and those extending beyond a month but under one year (n = 73, 31.7%). Only a small minority (n = 7, 3.0%) experienced drug shortages that persisted for more than a year, indicating that while extended disruptions are rare, they are not negligible.

When asked about the time required to obtain an equivalent alternative for a non-available medication, 31.7% of respondents (n = 73) reported that they could find a substitute on the same day (Table 2). A comparable proportion indicated challenges: 30.9% (n = 71) found it difficult to locate an alternative within a reasonable timeframe, while 30.0% (n = 69) were able to do so within a week. Notably, 7.4% (n = 17) were unable to find any suitable substitute, reflecting critical access barriers for certain medications.

In response to drug shortages, participants could select more than one action taken. The most common action reported among 230 respondents was to prescribe an alternative medication, selected by 77.4% of participants (n = 178). Additionally, some respondents advised patients to wait for the drug to become available (n = 81, 35.2%) or to search for it themselves (n = 77, 33.5%). Contacting other healthcare facilities to source medications was practiced by 22.6% (n = 52). A small subset (n = 15, 6.5%) selected “Other” as their course of action, suggesting diverse or situationally dependent strategies (Table 2).

Participants reported shortages across several medication categories in a multiple-response question, where each respondent could select several options (Table 3). The most frequently affected were anti-infective (n = 84, 36.5%) and analgesics/pain medications (n = 82, 35.7%), followed closely by cardiovascular and anti-seizure medications (≈27%). Notably, a substantial number of respondents also identified shortages in immunomodulators, antihypertensives, and anti-diabetic medications (≈20–25%). Other commonly affected categories included ophthalmic, vaccines, and transplant medications (≈18%), while psychiatric, oncology, and anti-coagulant drugs were less frequently reported (<16%). Reproductive system medications were the least affected (4.8%).

During the matched 12-month period, SFDA shortages were most frequently categorized as Other (35.3%), defined as entries that could not be confidently mapped to any of the predefined therapeutic classes of this study based on the available product/active-ingredient information. This was followed by anti-infective medications (14.0%) and antihypertensive medications (11.8%). Moderate SFDA representation was observed for anti-diabetic medications (6.6%), antipsychotic medications (6.6%), and analgesics/pain medications (5.1%), whereas ophthalmic medications, vaccines, anti-hepatitis medications, transplant medications, and reproductive system medications had 0.0% SFDA-listed shortages during the same period (Table 3).

Participants were asked an open-ended question: “If you recall the name of the medication that you have encountered a shortage of, kindly specify.” Analysis of these free-text responses revealed a broad and diverse pattern across therapeutic classes.

Filgrastim, an immunomodulatory agent, was the most frequently cited medication, reported by seven respondents (3.7%). Lorazepam (an anxiolytic) and acetaminophen (an analgesic) were each reported by six respondents (3.2%). Other frequently cited medications included melatonin (a hormone supplement) and hydromorphone (an opioid analgesic), each mentioned by four respondents (2.1%). Medications cited by three respondents (1.6%) included Movicol^®^ (a laxative), potassium phosphate (an electrolyte supplement), and tacrolimus (an immunosuppressant used in transplant patients).

#### 3.1.3. Impact on Patient Care

Among the 208 participants who were prescribed an alternative medication during a drug shortage, 55.3% (n = 115) reported that the alternative was adequately effective compared to the originally intended therapy (Table 4). However, 30.8% (n = 64) indicated that the alternative medication was not as effective as the original, potentially impacting patient outcomes. Additionally, 13.9% (n = 29) of respondents noted that patients refused the alternative medication, further complicating continuity of care.

Drug shortages were associated with a variety of negative consequences within healthcare facilities. Participants could select more than one reported consequence. The most frequently reported consequence was delayed care, cited by 44.0% (n = 159) of respondents (Table 4). Other significant impacts included higher healthcare costs (n = 74, 20.5%), an increase in hospitalization rates (n = 63, 17.5%), and complications following the use of alternative medications (n = 55, 15.2%). A smaller group (n = 10, 2.8%) reported other consequences, reflecting a wide array of facility-level challenges arising from drug shortages.

#### 3.1.4. Reporting to the SFDA

Table 5 summarizes healthcare professionals’ awareness and engagement with drug-shortage management and reporting systems. Nearly half of the respondents (46.1%, n = 106) reported that their healthcare facilities had a standard procedure in place for managing drug shortages. However, a substantial proportion (41.3%, n = 95) were uncertain whether such procedures existed, and 12.6% stated that no standard procedure was available.

Regarding familiarity with the Saudi Food and Drug Authority (SFDA) drug-shortage reporting system, most participants (71.7%, n = 165) indicated they were not familiar with the reporting system, while only 28.3% (n = 65) reported awareness of its use. Correspondingly, very few respondents (8.3%, n = 19) had personally reported a drug shortage to the SFDA, whereas the vast majority (91.7%, n = 211) had never submitted a report.

Responses regarding SFDA feedback varied among the 230 healthcare professionals. Only 5.7% (n = 13) reported receiving a timely response, 2.2% (n = 5) noted delays, and another 5.7% (n = 13) received no response at all. The remaining 86.5% (n = 199) selected “not applicable,” reflecting limited engagement with the reporting system overall.

#### 3.1.5. Duration and Notification of Shortages

**Figure 2:**
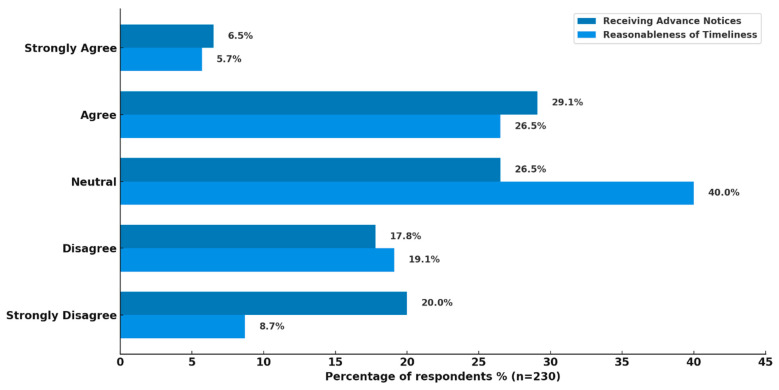
Perceptions of healthcare professionals regarding advance notice and resolution timeliness of drug shortages.

All healthcare professionals (n = 230) responded to this item, with respondents expressing mixed views regarding advance notification of potential drug shortages (Figure 2). The responses were varied, reflecting differing experiences across healthcare settings. A plurality of participants agreed that they received advance notice, 29.1% (n = 67), while 26.5% (n = 61) remained neutral. Conversely, a considerable proportion reported the opposite experience: 17.8% (n = 41) disagreed and 20.0% (n = 46) strongly disagreed, suggesting that many healthcare professionals do not feel adequately informed ahead of shortages. Only a small minority (6.5%, n = 15) strongly agreed with receiving advance notifications.

Participants also rated the reasonableness of the time taken to resolve drug shortages (Figure 2). Most respondents (40.0%, n = 92) were neutral, reflecting uncertainty or variability in experiences. Among those who expressed a clear opinion, 26.5% (n = 61) agreed that the resolution time was reasonable, whereas 19.1% (n = 44) disagreed, and 8.7% (n = 20) strongly disagreed. Only 5.7% (n = 13) of respondents strongly agreed, indicating that perceptions of timely resolution remain limited overall.

#### 3.1.6. Documentation of Shortages

**Figure 3:**
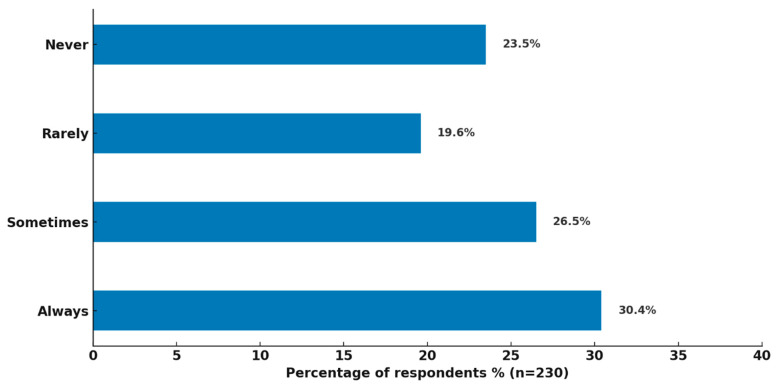
Frequency of documenting drug shortages among healthcare professionals.

All healthcare professionals (n = 230) responded to this item regarding how frequent drug shortages are documented in their healthcare setting. The most common response was “Always,” reported by 30.4% of respondents (n = 70), suggesting that nearly one-third of facilities maintain consistent documentation practices (Figure 3).

However, a sizable proportion documented shortages only sometimes (26.5%, n = 61), rarely (19.6%, n = 45), or never (23.5%, n = 54). Collectively, this means that nearly 70% of respondents reported either inconsistent or no documentation at all.

#### 3.1.7. Causes and Solutions of Drug Shortages

**Figure 4:**
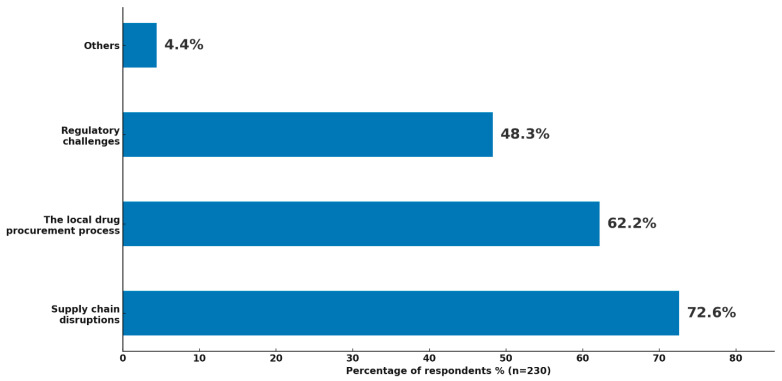
Factors perceived to contribute to drug shortages in healthcare settings.

All respondents (n = 230) identified several factors contributing to drug shortages within their healthcare settings (Figure 4). The most frequently reported cause was supply chain disruptions (72.6%, n = 167), highlighting the widespread impact of logistical challenges on drug availability. This was followed by issues related to the local drug procurement process (62.2%, n = 143), suggesting that internal administrative inefficiencies and sourcing delays are also major contributors. Regulatory challenges (48.3%, n = 111) were additionally cited, reflecting the influence of national or institutional policies and compliance barriers on timely medication access. A small proportion of respondents (4.4%, n = 11) mentioned other factors, including financial constraints, workforce shortages, and supplier limitations.

**Figure 5:**
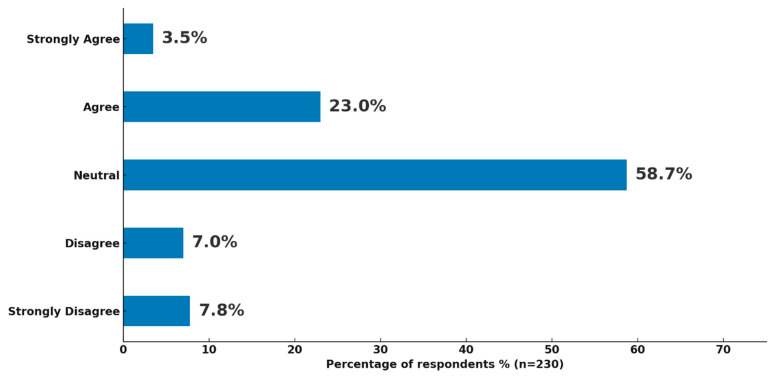
Perceived effectiveness of implemented strategies to mitigate drug shortages.

Respondents (n = 230) were asked to evaluate the effectiveness of existing strategies implemented in their institutions to reduce drug shortages (Figure 5). More than half of the participants (58.7%, n = 135) selected a neutral response, suggesting uncertainty or limited awareness regarding the outcomes of these strategies. Among those with a positive perception, 23.0% (n = 53) agreed, and 3.5% (n = 8) strongly agreed that the implemented strategies were effective. In contrast, 7.0% (n = 16) disagreed, and 7.8% (n = 18) strongly disagreed, indicating that only a small minority perceived the current strategies as ineffective.

In response to the open-ended question, “In your opinion, what effective strategies can be implemented to minimize drug shortages?”, healthcare professionals proposed a diverse range of practical and system-level interventions. Qualitative analysis of their responses revealed several themes.

The most frequently cited strategy was to enhance supply chain management. Respondents emphasized the need for continuous monitoring of supply vulnerabilities, timely ordering of medications, and maintaining optimal stock levels to prevent depletion. Many recommended developing priority lists for essential or life-saving medications, alongside identifying therapeutic alternatives that could be readily substituted during shortages.

Communication and coordination were highlighted as critical elements for effective shortage mitigation. Suggestions included implementing automated restocking alerts, real-time drug tracking systems across departments, and keeping healthcare teams and patients informed about available alternatives and shortage status.

Another theme centered on improved inventory planning and forecasting, particularly for high-demand medications. Participants highlighted the need for frequent stock assessments, better demand prediction models, and diversification of suppliers to reduce dependence on single sources and enhance system resilience.

A subset of participants proposed regulatory and policy reforms. These included enabling direct procurement from manufacturers, considering privatization of pharmaceutical supply chains, and reinforcing oversight of procurement and distribution practices to increase efficiency and responsiveness.

Lastly, respondents emphasized the need for inter-facility collaboration. Strategies included resource sharing, prompt reporting of shortages to regulatory authorities, and cross-institutional coordination to manage and redistribute supplies more effectively during critical shortages. Collectively, these insights underscore a strong awareness among healthcare professionals of the multifactorial nature of drug shortages and the systemic interventions required to address them.

### 3.2. Healthcare Users

#### 3.2.1. Demographic Characteristics

A total of 243 participants completed the survey (Table 6). The largest age group was 25–34 years (22.6%), followed by 45–54 years (19.3%), 35–44 years (17.7%), and 55–64 years (15.6%). Younger adults aged 18–24 years (13.2%) and those 65 years or older (11.5%) comprised smaller proportions of the sample. The majority of respondents were female (71.3%), a significantly higher proportion than for males (28.3%). The number of respondents varied slightly across items due to non-response or conditional questions. A Chi-square goodness-of-fit test comparing this distribution to an expected value yielded a statistically significant difference (*p* < 0.001), indicating a significantly higher proportion of female respondents.

In response to the open-ended survey question inviting participants to disclose their current medical diagnoses or health concerns, a total of 150 responses were analyzed. The results revealed a wide spectrum of health conditions, underscoring the heterogeneity in the participants’ health profiles.

The most frequently reported condition was diabetes, cited by 21.3% of respondents. This was closely followed by hypertension or blood pressure-related issues, reported by 19.3%. Thyroid disorders emerged as the third most prevalent concern, accounting for 10.7% of responses, while heart conditions were reported by 8.7% of participants.

Several other recurring health concerns were also noted. Vitamin deficiencies were reported by 6.0%, followed by anemia or other blood-related disorders and liver issues, both at 3.3%. Mental health concerns and rheumatism were each mentioned by 2.7% of respondents. Lastly, cholesterol-related problems appeared in 2.0% of the submissions. These findings indicate a predominance of chronic, non-communicable diseases, with metabolic and cardiovascular conditions being particularly prominent among the surveyed population.

#### 3.2.2. Experience with Drug Shortages

Participants were asked about their experiences with essential drug shortages over the previous two years (Table 7). Of the 243 respondents, 30.9% reported experiencing shortages or the unavailability of essential medications, while 69.1% reported no such experience. Among the 242 respondents to the question regarding the drug shortage in the past year, most participants (69.4%) stated they never experienced shortages, whereas 24.4% experienced them occasionally and 6.2% reported shortages occurring very often. Although most respondents did not experience regular shortages, a notable minority reported interruptions in medication availability.

Participants (n = 242) also rated their satisfaction with the availability of their prescribed medications at their pharmacy using a 5-point Likert scale (1 = very dissatisfied; 5 = very satisfied). Overall satisfaction was high: 64% were very satisfied (score 5) and 23.2% selected score 4, indicating moderate-to-high satisfaction. Lower satisfaction levels were less frequent, with 9.5% selecting score 3, 2.5% selecting score 2, and 0.8% selecting score 1. Nearly 87% reported moderate-to-high satisfaction, indicating generally good access at the pharmacy level.

Among the 57 respondents who answered the open-ended question on specific drug shortages, the responses were reviewed and grouped into therapeutic drug classes by the research team and summarized by frequency of mention. Of these, 26.3% reported no shortages. However, a notable proportion of participants identified specific medications or categories that were difficult to obtain.

Reported shortages most commonly involved diabetes medications (7.0%), which included insulin and oral hypoglycemics, and unspecified antibiotics (5.3%). Blood pressure medications, cholesterol drugs, and vitamins/supplements were each cited by 3.5%.

Several individual medications, each mentioned once (1.8%), included Creon^®^ (pancreatic enzyme), nasal sprays, metronidazole (antiprotozoal drug), topical creams, Acyclovir (antiviral agent), filgrastim (granulocyte-colony stimulating factor [GCSF]), azithromycin (antibacterial agent), Nexium^®^ (proton pump inhibitor [PPI]), hypertonic saline irrigation, Roaccutane^®^ (retinoid anti-acne agent), levothyroxine, magnesium oxide, Humira^®^ (tumor necrosis factor [TNF] inhibitor), oral contraceptives, and several others. Some responses were not specific, such as “not remembered” or “all available.”

Overall, while many participants did not encounter shortages, a diverse range of essential medications were reported as unavailable; chronic disease treatments (especially for diabetes and cardiovascular conditions) were the most prominent.

#### 3.2.3. Access to Alternative Medications

**Figure 6:**
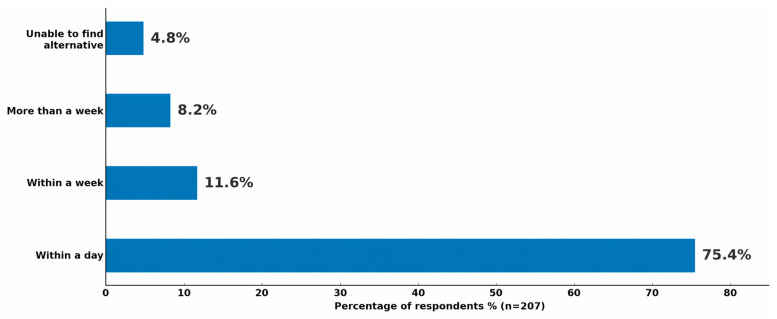
Time required to find an alternative medication source.

Among those who reported being prescribed an alternative due to a shortage, most were able to secure a substitute in a timely manner (Figure 6). Specifically, 75.4% obtained an alternative within one day, 11.6% within a week, and 8.2% after more than a week. However, 4.8% were unable to find an alternative medication at all. These results indicate that while timely substitution is generally achievable, a notable minority still experience delays or unmet needs, which could compromise treatment continuity and health outcomes.

Participants who had received alternative medications due to shortages were asked to evaluate both the quality of these substitutes and their confidence in these medications’ safety and effectiveness (Table 8). Regarding perceived quality, among the 242 respondents, the majority (57.9%) rated alternatives as having a similar quality to their usual prescriptions. A smaller proportion (15.7%) perceived the alternatives as higher in quality, while 14.0% and 12.4% rated them as much lower and lower in quality, respectively.

In terms of confidence in the safety and effectiveness of these alternatives, 26.0% of the 242 respondents were extremely confident, and 23.1% were very confident. The largest group (28.9%) expressed moderate confidence, while 7.0% were slightly confident, and 14.9% reported being not confident at all.

These findings suggest that while a majority of participants accepted the quality of alternative medications as comparable to their usual treatments, a substantial minority harbored concerns, particularly regarding safety and effectiveness, which may influence adherence and satisfaction.

#### 3.2.4. The Impact of Medicine Shortages on Public Health

Participants were asked to evaluate the overall impact of drug shortages on their health. Over half of the 242 respondents (53.3%) reported that shortages had no impact on their health (Table 9). However, a notable subset indicated adverse effects, with 16.5% reporting a significant impact, 15.3% a minor impact, and 14.9% a moderate impact.

When asked to describe how shortages had affected them personally (selecting all that applied), 45.3% reported no specific adverse effects. Among those affected, the most common issues included increased anxiety or stress (15.3%), inability to afford alternative medications (12.3%), and worsening of their medical condition (11.7%). Other reported outcomes included experiencing more adverse effects from substitute medications (10.8%) and hospitalization due to the unavailability of standard treatments (4.5%).

These results highlight that although a majority experienced no direct health impact, a substantial proportion of respondents encountered both clinical and psychological consequences related to drug shortages.

#### 3.2.5. Financial Impact

Participants were asked whether they incurred additional costs as a result of drug shortages, such as purchasing a more expensive brand, sourcing medications from other providers, or traveling to alternate pharmacies (Table 10). The majority (71.9%) reported no additional financial burden, while 28.1% indicated that they had incurred extra costs due to these shortages.

Among the 67 who reported additional expenditure, most (53.7%) estimated their expenses to be between SAR 201 and 500. Another 37.3% reported spending less than SAR 200, 7.5% incurred costs between SAR 501 and 1000, and a small minority (1.5%) spent more than SAR 1000. Although most participants did not face financial repercussions, a notable proportion experienced varying degrees of economic burden when managing drug shortages.

#### 3.2.6. Overall Perception and Recommendations

Participants were asked about their understanding of the causes behind drug shortages. Among the 242 respondents, the majority (65.3%) reported not being informed at all (Table 11). An additional 16.5% felt slightly informed, and 9.9% considered themselves moderately informed. Only a small portion of respondents described themselves as very informed (2.9%) or extremely informed (5.4%).

Regarding awareness of the SFDA’s drug-shortage reporting system, only 13.2% of the 242 participants were aware of its existence, while 86.8% had no prior knowledge of the system. Actual reporting of drug shortages to the SFDA was even more limited, with only 1.7% having submitted a complaint or report, while the vast majority (98.3%) had never reported any shortages. When asked whether they had received a response from the SFDA regarding their report, 98.8% of the 241 respondents marked the question as not applicable, reflecting the low reporting rate. Among the four participants who reported drug shortages to the SFDA, one respondent received a timely response, two reported receiving no response, and one respondent did not answer the follow-up question.

These findings reveal a considerable gap in public awareness, engagement, and responsiveness regarding official channels for reporting drug shortages, which may impede effective communication and timely resolution of supply issues.

## 4. Discussion

The findings from this study underscore the pervasive issue of drug shortages within the healthcare system, highlighting significant implications for patient care and the operational efficacy of healthcare facilities. With over 89% of healthcare professionals reporting at least one drug-shortage incident in 2024, and nearly 40% encountering shortages more than ten times, it is evident that this challenge is not isolated but rather systemic [1]. The demographic profile of participants, predominantly female and concentrated in the 30–39 age group, reflects a workforce that is more likely to experience the brunt of these shortages, given their frontline roles in patient care [18].

The data reveal that drug shortages are not only frequent but also varied across therapeutic classes. Anti-infective and analgesic medications topped the list, affecting critical areas of care and potentially compromising treatment outcomes for patients with infections or pain [19]. The significant proportion of respondents indicating that they could not find suitable alternatives within a reasonable timeframe (30.9% reported difficulty) raises concerns about continuity of care and the potential for adverse patient outcomes [20]. This aligns with existing literature that suggests drug shortages can lead to increased hospitalization rates and complications, as healthcare providers struggle to manage patients with suboptimal alternatives [19].

The SFDA benchmark provides an important national reference point for interpreting hospital-reported shortages. The concentration of SFDA-listed shortages in major therapeutic areas, particularly anti-infectives and antihypertensives, supports the view that SFDA surveillance is capturing clinically relevant supply pressures and can serve as an early warning signal for categories likely to affect hospital operations.

However, discrepancies between hospital-reported shortages and SFDA-listed shortages carry important practical implications. When a drug class is frequently reported as unavailable at the hospital level but is not reflected in SFDA shortage listings, this suggests the problem may originate from internal factors, such as procurement cycles, formulary management, or inventory distribution, rather than a true national supply disruption. Conversely, when SFDA-listed shortages are not prominently reported at the hospital level, it may indicate that the institution has developed effective mitigation strategies, such as therapeutic substitution protocols or buffer stock policies, that are buffering the national shortage from reaching the patient. Understanding the direction and pattern of these discrepancies is therefore critical: they can help hospital administrators to distinguish between system-level failures requiring regulatory intervention and facility-level gaps addressable through internal procurement and inventory reforms [21].

The qualitative analysis of open-ended responses reflects the nature of drug shortages across diverse therapeutic classes. Filgrastim, a critical immunomodulatory agent in oncology, was the most frequently mentioned, aligning with recent literature that identifies cancer care as particularly vulnerable due to the complex supply chain and high reliance on active pharmaceutical ingredients (APIs). For instance, Adeyemo and Bunmi (2025) found that oncology drug shortages are increasingly driven by disruptions in API supply and lapses in Good Manufacturing Practices, leading to significant barriers in consistent treatment delivery [22].

The reported shortages of Lorazepam and Acetaminophen extend the impact beyond oncology into mental health and primary care, reflecting what Janetzki et al. (2025) documented in their nationwide survey of Australian pharmacists: widespread shortages of everyday medications not only disrupted workflows but also burdened pharmacists with increased substitution tasks and administrative duties [12]. These findings resonate with the current data showing that both acute (e.g., pain management) and chronic (e.g., anxiety disorders) treatment regimens are being undermined by erratic availability.

The implications of drug shortages on patient care are profound. While over half of the respondents perceived alternative medications to be adequately effective, nearly 31% indicated that alternatives were less effective than intended therapies, which could adversely affect patient outcomes [23]. These findings reflect respondents’ perceptions and should not be interpreted as evidence of comparative clinical efficacy. Furthermore, the 44% of respondents reporting delayed care as a consequences of drug shortages underscore a critical gap in healthcare delivery, where timely access to medications is paramount for effective treatment [24]. The variability in patient responses to alternative medications, with 13.94% refusing substitutes, further complicates the situation, suggesting that patient preferences and perceptions play a significant role in treatment adherence and satisfaction [25].

This study also highlights significant gaps in the reporting of drug shortages to the Saudi Food and Drug Authority (SFDA). With 91.7% of respondents indicating that they had never reported a shortage, there is a clear disconnect between the incidence of shortages and the formal mechanisms in place for addressing them [4]. The lack of familiarity with the SFDA’s reporting system among 71.7% of respondents points to a critical barrier that hinders effective communication and response to drug shortages [25]. This gap in awareness and engagement may limit the SFDA’s ability to track and respond to supply disruptions, ultimately jeopardizing patient safety, and care quality.

Similar gaps were observed in patient awareness and engagement with regulatory reporting systems, mirroring findings in European and North American contexts [26]. With only 1.7% of participants reporting shortages to the SFDA and over 85% unaware of the reporting system, there is an evident disconnect between policy frameworks and real-world implementation. In addition, this study highlights a significant gap in public understanding and utilization of formal mechanisms to address drug shortages. A substantial majority of respondents (65.3%) reported being uninformed about the causes of drug shortages, and only 13.2% were aware of the SFDA’s drug-shortage reporting system, further indicating severe underutilization of patient-centered pharmacovigilance tools.

These findings are consistent with European studies showing that patients are often unaware of formal reporting channels or lack confidence in their efficacy [27]. Similar gaps were observed in Portugal; although 52.2% of patients had experienced a drug shortage, awareness and utilization of reporting systems remained limited [28]. Bridging this gap requires targeted public education, streamlined reporting processes, and improved feedback mechanisms to enhance engagement with regulatory reporting systems and strengthen national drug-shortage surveillance.

In conclusion, the findings demonstrate that limited awareness, low engagement with formal reporting systems, and inadequate communication affect both healthcare professionals and healthcare users, resulting in underreporting of drug shortages and weakening coordinated regulatory responses.

Participants identified several factors contributing to drug shortages, with supply chain disruptions and local procurement processes being the most frequently cited. These findings align with existing literature that emphasizes the complexity of drug supply chains and the multifaceted or numerous challenges they face, from manufacturing delays to regulatory hurdles [1]. In a five-year hospital-based analysis, drug shortages were primarily driven by regulatory issues and manufacturing delays (39%), followed by unknown causes (29%) and supply chain disruptions (10%), thereby highlighting the convergence of regulatory, production, and logistical factors shaping shortage patterns within hospital settings [29]. The suggestions for enhancing supply chain management, improving communication, and fostering inter-facility collaboration reflect a comprehensive understanding of the multifaceted nature of this issue [30]. Consistent with these complexities, our results demonstrate substantial variability in healthcare providers’ ability to manage shortages, with roughly one-third securing medication alternatives promptly, another third experiencing significant delays, and a critical minority unable to find suitable substitutes. These findings indicate that while most healthcare providers attempt proactive management of shortages through substitutions or referrals, a non-negligible number resort to patient-driven solutions or passive waiting strategies, which may compromise treatment continuity or patient safety. Moreover, the questionnaire responses regarding communication about impending drug shortages and perceptions of resolution timelines reveal critical insights into systemic inefficiencies and perceived variability across healthcare settings. These results suggest a degree of uncertainty or variability in experiences, as reflected by the high neutral response rate.

Although more participants agreed than disagreed that resolution times are reasonable, a significant portion were dissatisfied; this highlights the need to improve transparency and communication regarding timelines for shortage resolution. The qualitative responses to the open-ended query on strategies for mitigating drug shortages revealed five interrelated themes: supply chain optimization, communication and coordination including automated alerts, real-time tracking systems, and dynamic dashboards to update clinicians on drug status. These findings are consistent with previous reports highlighting the importance of timely information sharing, supply chain visibility, and coordinated stakeholder communication in reducing the impact of drug shortages.

Moreover, the call for regulatory reforms and direct procurement from manufacturers indicates a recognition that systemic changes are necessary to mitigate shortages effectively [23]. These insights align with broader discussions in the field regarding the need for more robust supply chain frameworks and better coordination among stakeholders to ensure the availability of essential medications.

Although over half of the participants in this study reported no health impact due to drug shortages, a notable 47% experienced varying degrees of adverse effects, including increased anxiety, disease worsening, and hospitalization. These results align with prior international findings, which noted that shortages can cause treatment delays, forced substitutions, and compromised health outcomes [27].

According to a study conducted in 2021, patients impacted by drug shortage experienced physiological distress from having to search for alternatives, worsened health conditions, delayed treatment, and higher out-of-pocket expenses [28].

Moreover, a 2021 study across six European hospitals found that although many patients did not initially perceive drug shortages as problematic, those directly affected especially in oncology and hematology, reported serious consequences such as health deterioration and treatment interruptions [26].

Approximately 28% of participants reported incurring additional expenses due to drug shortages. While most affected individuals spent SAR 201–500, some reported significantly higher costs, indicating variability in the financial burden experienced by patients. A similar financial impact has been documented in the United States by a report to Congress highlighting the impact of drug shortages on consumer costs. The financial burden on the patients was attributed to several factors, including increased prices of the shortage medications, higher costs of alternative medications, and indirect expenses such as travel, time burden, and the cost of multiple medical appointments [31].

Nearly half of participants reported being prescribed alternative medications due to the unavailability of their usual treatment. While most rated the substitutes as similar or better in quality, a substantial minority (26.4%) believed the alternatives were inferior. Confidence in the safety and efficacy of these alternatives was also mixed, with 21.9% of respondents expressing low to no confidence.

These perceptions are clinically relevant, as negative beliefs about medication quality may reduce adherence and therapeutic effectiveness. Previous research supports this concern, emphasizing the need for transparent communication and regulatory assurance about substitute medication standards [27]. A 2023 study reported that a substantial percentage of participants expressed a distrust towards generic medications compared to branded ones, in terms of quality, safety and efficacy, which indicates the need for targeted educational initiatives [32].

Although this study has revealed interesting results in exploring the consequences of essential medicine shortages on healthcare professionals and users, several limitations must be acknowledged. This study was conducted within a single institution which may limit the ability to generalize the findings to other healthcare institutions. The relatively short data collection period may have influenced participant recruitment and sample representativeness. In addition, the cross-sectional design prevents us from establishing causal relationships or temporal trends. The use of convenience sampling may introduce selection bias, as the sample may not be fully representative of the broader population. Furthermore, reliance on self-reported data may introduce recall bias. Variability in the recall period across survey items may also affect comparability with SFDA-reported data. Finally, the findings reflect perceived experiences and were not verified using data sources such as inventory records or regulatory shortage databases.

## 5. Conclusions

This study highlights the need to address drug shortages as a systemic challenge with substantial perceived clinical, economic, and operational consequences. The widespread frequency and cross-therapeutic nature of shortages, particularly affecting critical drug classes such as anti-infectives, cardiovascular medications, anti-diabetics, and analgesics, were perceived by respondents as posing direct risks to treatment continuity and patient care. Consistent with global findings, a significant portion of healthcare users reported experiencing delayed care, perceived reductions in medication efficacy, and heightened psychological stress, reflecting subjective assessments of how shortages affect care quality rather than objectively verified clinical outcomes. These perception-based findings nonetheless carry important clinical relevance, as negative experiences and low confidence in alternative medications may reduce adherence and undermine trust in healthcare systems. Finally, this study underscores the importance of supply chain resilience, inter-facility coordination, and regulatory engagement. The observed discrepancies between hospital-reported shortages and SFDA national data suggest gaps in current reporting mechanisms. Enhancing integration between hospital-level inventory systems and national monitoring platforms, potentially through digital and automated reporting approaches, may improve the accuracy, timeliness, and completeness of shortage reporting, supporting more proactive management and improved patient care.

## Figures and Tables

**Table 1:** Characteristics of the study participants.

Variable	Frequency (n)	Percentage (%)
**Gender**		
Male	70	30.4
Female	160	69.6
**Age category**		
Less than 30	66	28.7
30–39	112	48.7
40–49	37	16.1
50–59	12	5.2
More than 60	3	1.3
**Years of experience**		
2 to 5 years	86	37.4
6 to 10 years	57	24.8
More than 10 years	87	37.8
**Current profession**		
Physician	26	11.3
Pharmacist	48	20.9
Nurse	94	40.9
Others	62	26.9

**Table 2:** Duration, management, and actions taken by healthcare professionals during essential drug shortages.

Variable	Frequency (n)	Percentage (%)
**Duration of essential drug shortages reported by healthcare professionals**		
One week or less	74	32.2
More than a week, less than a month	76	33
More than a month less than a year	73	31.7
More than a year	7	3
**Time to find equivalent alternative for non-available medication**		
Same day	73	31.7
Difficult to find an equivalent alternative within a reasonable time	71	30.9
Within a week	69	30.0
Unable to find an equivalent alternative within a reasonable time	17	7.4
**Action taken during drug shortage**		
Prescribe alternative medication	178	77.4
Contact other healthcare facilities	52	22.6
Advise patient to search for the medication	77	33.5
Advise patient to wait until the drug becomes available	81	35.2
Other	15	6.5

**Table 3:** Respondent reported drug shortages by therapeutic class and comparison with SFDA-listed shortages.

Type of Medication Facing Shortage	Healthcare Professional–Reported Shortages (n)	Percentage (%)	SFDA-Listed Shortages (n)	Percentage (%)
Anti-infective medications	84	36.5	19	14
Analgesics and pain medications	82	35.7	7	5.1
Cardiovascular medications	63	27.4	3	2.2
Anti-seizure medications	62	27	4	2.9
Immunomodulator medications	57	24.8	2	1.5
Antihypertensive medications	53	23	16	11.8
Anti-diabetic medications	45	19.6	9	6.6
Ophthalmic medications	43	18.7	0	0
Vaccines	43	18.7	0	0
Transplant medications	41	17.8	0	0
Anti-inflammatory medications	40	17.4	5	3.7
Dermatology medications	37	16.1	3	2.2
Antipsychotic medications	36	15.7	9	6.6
Antineoplastic medications	34	14.8	5	3.7
Antiallergic and anaphylaxis medications	33	14.3	2	1.5
Anti-coagulation medications	31	13.5	3	2.2
Anti-hepatitis medications	26	11.3	0	0
Anesthetics and pre-operative medications	24	10.4	1	0.7
Reproductive system medications	11	4.8	0	0
Other	40	16.2	48	35.3

Note: Survey-reported data reflects healthcare professionals’ perceived frequency of drug shortages within the study setting, whereas SFDA data represent officially reported national shortages. These measures are based on different data sources and methodologies and are not directly comparable.

**Table 4:** Effectiveness of alternative medications and reported consequences of drug shortages.

Variable	Frequency (n)	Percentage (%)
**In case of prescribing an alternative medication**		
The alternative was adequately effective	115	55.3
The alternative was not effective as the original	64	30.8
Patient refused the alternative medication	29	13.9
**Common consequences of drug shortages in your facility**		
Delayed care	159	44.0
Higher cost	74	20.5
Increase hospitalization	63	17.5
Complication after using alternative medications	55	15.2
Other	10	2.8

**Table 5:** Awareness, familiarity, and reporting behavior of healthcare professionals regarding drug shortages and the SFDA reporting system.

Variable	Frequency (n)	Percentage (%)
**Awareness of standard procedures for managing drug shortages in healthcare facilities**		
Yes	106	46.1
No	29	12.6
I don’t know	95	41.3
**Familiarity with the SFDA drug-shortage reporting system**		
Yes	65	28.3
No	165	71.7
**Experience of healthcare professionals in reporting drug shortages to the SFDA**		
Yes	19	8.3
No	211	91.7
**Response from the SFDA regarding reported cases**		
Delayed response	5	2.2
Timely response	13	5.7
No response	13	5.7
Not applicable	199	86.5

**Table 6:** Demographic distribution of survey respondents by age and gender.

Variable	Frequency (n)	Percentage (%)
**Gender**		
Female	174	71.6
Male	69	28.4
**Age**		
25–34	55	22.6
45–54	47	19.3
35–44	43	17.7
55–64	38	15.6
18–24	32	13.2
More than 65	28	11.5

**Table 7:** Participants’ experiences, frequency, and satisfaction related to essential drug shortages and availability.

Variable	Frequency (n)	Percentage (%)
**Experience of essential drug shortages in the past two years**		
No	168	69.1
Yes	75	30.9
**Reported frequency of prescribed drug shortages over the past year**		
Never	168	69.4
Sometimes (once every few months)	59	24.4
Very often	15	6.2
**Satisfaction with availability of prescribed medications**		
Very dissatisfied	2	0.8
Dissatisfied	6	2.5
Neutral	23	9.5
Satisfied	56	23.2
Very satisfied	155	64.0

**Table 8:** Perceived quality and confidence in alternative medications.

Variable	Frequency (n)	Percentage (%)
**Perceived quality of alternative medications compared to usual prescriptions**		
Similar quality	140	57.9
Higher quality	38	15.7
Much lower quality	34	14.0
Lower quality	30	12.4
**Confidence in the safety and effectiveness of alternative medications**		
Extremely confident	63	26.0
Very confident	56	23.1
Moderately confident	70	28.9
Slightly confident	17	7.0
Not confident at all	36	14.9

**Table 9:** Health impact and patient-reported effects of drug shortages.

Variable	Frequency (n)	Percentage (%)
**Evaluate the impact of drug shortages on overall health**		
No impact	129	53.3
Significant impact	40	16.5
Minor impact	37	15.3
Moderate impact	36	14.9
**Self-reported effects of drug shortages on patients**		
No effect	151	45.3
Increase anxiety or stress	51	15.3
Unable to afford the alternatives	41	12.3
Condition worsened	39	11.7
More adverse effects	36	10.8
Needed hospitalization	15	4.5

**Table 10:** Financial impact of drug shortages.

Variable	Frequency (n)	Percentage (%)
**Did you incur additional costs due to the shortage (e.g., buying a more expensive brand, ordering from another source, or traveling to other pharmacies)?**		
No	174	71.9
Yes	68	28.1
**If yes, what were the additional expenses for you (approximate amount)?**		
Less than SAR 200	25	37.3
SAR 201–500	36	53.7
SAR 501–1000	5	7.5
More than SAR 1000	1	1.5

**Table 11:** Awareness and engagement with the SFDA regarding drug shortages.

Variable	Frequency (n)	Percentage (%)
**Participant understanding of the causes of drug shortages**		
Not informed at all	158	65.3
Slightly informed	40	16.5
Moderately informed	24	9.9
Extremely informed	13	5.4
Very Informed	7	2.9
**Awareness of the SFDA drug-shortage reporting system**		
No	210	86.8
Yes	32	13.2
**Participant experience with reporting drug shortages to the SFDA**		
No	238	98.3
Yes	4	1.7
**Have you received any response from the SFDA regarding the drug shortages you reported?**		
Not applicable	238	98.8
Yes, in a timely manner	1	0.4
No response	2	0.8
Yes, but received a delayed response	0	0.0

## Data Availability

Data is contained within the article. Further inquiries can be directed to the corresponding author.

## Appendix A. Healthcare Professionals’ Questionnaire on the Impact of Medication Shortages in Saudi Hospitals

**“We are conducting a study to understand the impact of the shortage of essential medicines on healthcare outcomes. The information collected will help improve the availability and accessibility of essential medicines.”**

**Disclaimer:** Your participation in this survey is optional, and your data will be used for research purposes only, with complete confidentiality. The researcher assumes no legal or medical responsibility for the interpretation of the questions or the results of the study. Participation means that you agree to the use of your information in this research anonymously, without mentioning your name or any identifying information.

**Important Notice for Participants:** The Pharmacy Care Department works to minimize the impact of medication shortages by providing safe alternatives when needed. They also use the “Wasfati” system to secure medications, ensuring treatment continuity and reducing harm due to unavailable drugs.

Please answer the following questions:

**Demographic Information**

1. **Age:**
  ○ Less than 30
  ○ 30–39
  ○ 40–49
  ○ 50–59
  ○ More than 60
2. **Gender:**
  ○ Male
  ○ Female
3. **Professional Title:**
  ○ Physician
  ○ Clinical pharmacist
  ○ Pharmacist
  ○ Nurse
  ○ Other (please specify):
4. **Years of Experience in Healthcare:**
  ○ 2–5 years
  ○ 6–10 years
  ○ More than 10 years

**Main Questions**

**Section 1: Frequency and Types of Shortages**

5. **How frequently did you face a drug shortage in your practice during the last two years (2023 and 2024)?**
  ○ None
  ○ 2 or less
  ○ 3–5 times
  ○ 6–10 times
  ○ More than 10 times
6. **What type(s) of medications have you faced shortage in. (choose all that apply)**
  ○ Anti-infective medications
  ○ Anti-hepatitis medications
  ○ Immunomodulator medications
  ○ Antiseizure medications
  ○ Cardiovascular medications
  ○ Antineoplastic medications
  ○ Analgesic and pain medications
  ○ Antidiabetic medications
  ○ Antihypertension medications
  ○ Anesthetic and pre- operative medications
  ○ Antiallergic and anaphylaxis medications
  ○ Anti-coagulation medications
  ○ Antipsychotics medications
  ○ Anti-inflammatory medications
  ○ Transplant medications.
  ○ Dermatology medications
  ○ Ophthalmic medications
  ○ Reproductive system medications
  ○ Vaccines
  ○ Others (please specify):
7. **If you recall the name of the medication that you have encountered shortage of kindly specify (optional):**
  **…………………………………………………………………………………………..**
8. **What actions do you usually take when you face a drug shortage? (choose all that apply)**
  ○ Prescribe alternative medication
  ○ Contact other health care facilities
  ○ Advise patient to search for the medication
  ○ Advise patient to wait until the drug becomes available
  ○ Other (please specify):…………………..
9. **How long does it usually take to find an equivalent alternative of the “non-available” medication (e.g., from another source or using a different brand)?**
  ○ Same day
  ○ Within a week
  ○ Difficult to find an equivalent alternative within a reasonable time
  ○ Unable to find an equivalent alternative within a reasonable time
10. **How long the drug shortage of essential medication last**
  ○ One week or less
  ○ More than a week and less than a month
  ○ More than a month and less than a year
  ○ More than a year

**Section 2: Impact on Patient Care**

11. **In case of prescribing an alternative medication**
  ○ The alternative was adequately effective
  ○ The alternative was not as effective as the original
  ○ Patient refused the alternative medication
12. **What are the common consequences of drug shortages in your facility? (choose all the common consequences that apply)**
  ○ Increase hospitalization
  ○ More adverse events with alternative medications
  ○ Lack of efficacy with the alternative (condition worsens)
  ○ Delayed care
  ○ Higher cost
  ○ Complications after using alternative medication
  ○ Others (please specify):

**Section 3: Reporting to SFDA:**

13. **Do you have a standard procedure in your facility to deal with drug shortage?**
  ○ Yes
  ○ No
  ○ I don’t know
14. **Are you familiar with Saudi FDA drug shortage reporting system?**
  ○ Yes
  ○ No
15. **Have you reported a drug shortage to SFDA:**
  ○ Yes
  ○ No
16. **Have you received any response from SFDA regarding your reported cases?**
  ○ Yes, timely response
  ○ Yes, delayed response
  ○ No response
  ○ Not applicable

**Section 4: Duration and Notification of Shortages**

17. **I usually receive advance notices of potential drug shortages in my hospital.**
  ○ Strongly disagree
  ○ Disagree
  ○ Neutral
  ○ Agree
  ○ Strongly agree
18. **I believe that the average duration to appropriately resolve drug shortages in my workplace is reasonable.**
  ○ Strongly disagree
  ○ Disagree
  ○ Neutral
  ○ Agree
  ○ Strongly agree

**Section 5: Documentation of Shortages:**

19. **How frequently do you document medication shortages in your institution?**
  ○ Always
  ○ Sometimes
  ○ Rarely
  ○ Never

**Section 6: Causes and Solutions for Drug Shortages**

20. **I believe that the following factors contribute to drug shortages (select all that apply):-**
  ○ The local drug procurement process
  ○ Regulatory challenges
  ○ Supply chain disruptions
  ○ Increased demand
  ○ Other (please specify): __________________________
21. **Strategies that have been implemented to minimize the impact of drug shortages are becoming more effective.**
  ○ Strongly disagree
  ○ Disagree
  ○ Neutral
  ○ Agree
  ○ Strongly agree
22. **In your opinion what effective strategies can be implanted to minimize drug shortage (optional)**
  (open-ended)

Closing Statement

Thank you for your participation in this survey. Your insights are valuable in understanding the impact of drug shortages on patient care and the healthcare system in Saudi Arabia.

## Appendix B. Healthcare Users’ Questionnaire on Assessing the Impact of Essential Medicines Shortages on Healthcare Outcomes in Saudi Hospitals

**“We are conducting a study to understand the impact of the shortage of essential medicines on healthcare users. The information collected will help improve the availability and accessibility of essential medicines.”**

**Disclaimer:** Your participation in this survey is optional, and your data will be used for research purposes only, with complete confidentiality and without any impact on your medical care. The researcher assumes no legal or medical responsibility for the interpretation of the questions or the results of the study. Participation means that you agree to the use of your information in this research anonymously, without mentioning your name or any identifying information.

**Important Notice for Participants:** The Pharmacy Care Department works to minimize the impact of medication shortages by providing safe alternatives when needed. They also use the “Wasfati” system to secure medications, ensuring treatment continuity and reducing harm due to unavailable drugs.

**Please answer the following questions:**

**Introduction Questions:**

1. **What is your age?**
  ○ 18–24
  ○ 25–34
  ○ 35–44
  ○ 45–54
  ○ 55–64
  ○ 65 or older
2. **What is your gender?**
  ○ Male
  ○ Female
3. **What is your current diagnosis (optional)?**
  ○ (please specify):……………………………………………………….

**Main Questions:**

**Section 1: Experience with Medication Shortages**

4. **Have you experienced a shortage (or unavailability) of any essential medication during the last two years?**
  ○ Yes
  ○ No
5. **What is(are) the name of the medication(s) that you encounter shortage of (optional)?**
  ○ (please specify):
6. **How frequently have you experienced shortages of your prescribed medications in the past year?**
  ○ Never
  ○ Sometimes (every few months)
  ○ Very often
7. **How satisfied are you with the availability of your prescribed medications at your pharmacy?**
  ○ Very dissatisfied
  ○ Dissatisfied
  ○ Neutral
  ○ Satisfied
  ○ Very satisfied

**Section 2: Access to Alternative Medications**

8. **Have you ever been prescribed an alternative medication due to a shortage of your usual medication?**
  ○ Yes
  ○ No
9. **How long did it take to find an alternative source of the medication (e.g., another pharmacy, different brand)?**
  ○ Same day
  ○ Within a week
  ○ More than a week
  ○ Unable to find an alternative
10. **How would you rate the quality of alternative medications you have been prescribed in comparison to your usual medications?**
  ○ Much lower quality
  ○ Lower quality
  ○ Similar quality
  ○ Higher quality
11. **How confident are you regarding the safety and effectiveness of the alternative medications?**
  ○ Not confident at all
  ○ Slightly confident
  ○ Moderately confident
  ○ Very confident
  ○ Extremely confident

**Section 3: Impact of Medication shortage on your overall health**

12. **How would you rate the impact of shortages in specific classes of medications on your overall health?**
  ○ No impact
  ○ Minor impact
  ○ Moderate impact
  ○ Significant impact
13. **How did the shortage affect you? (Check all that apply)**
  ○ Condition worsened
  ○ New side effects
  ○ Increased anxiety/stress
  ○ Needed hospitalization
  ○ Could not afford the alternatives
  ○ Other (please specify): ____________

**Section 4: Financial Impact**

14. **Did you incur additional costs due to the shortage (e.g., buying a more expensive brand, ordering from another source, or traveling to other pharmacies)?**
  ○ Yes
  ○ No
15. **If yes, what were the additional expenses for you (approximate amount)?**
  ○ Less than 200 SAR
  ○ 200–500 SAR
  ○ 501–1000 SAR
  ○ More than 1000 SAR

**Section 5: Overall Perception and Recommendations**

16. **How informed do you feel about the reasons behind medication shortages?**
  ○ Not informed at all
  ○ Slightly informed
  ○ Moderately informed
  ○ Very informed
  ○ Extremely informed
17. **Are you familiar with Saudi FDA drug shortage reporting system?**
  ○ Yes
  ○ No
18. **Have you reported a drug shortage to SFDA:**
  ○ Yes
  ○ No
19. **Have you received any response from SFDA regarding your reported cases?**
  ○ Yes, timely response
  ○ Yes, delayed response
  ○ No response
  ○ Not applicable

Thank You!

We appreciate your time and insight in completing this questionnaire. Your feedback will help us understand the challenges patients face and support improvements in the availability of essential medicines.
